# Alcohol Production as an Adaptive Livelihood Strategy for Women Farmers in Tanzania and Its Potential for Unintended Consequences on Women’s Reproductive Health

**DOI:** 10.1371/journal.pone.0059343

**Published:** 2013-03-19

**Authors:** Sandra I. McCoy, Lauren J. Ralph, Wema Wilson, Nancy S. Padian

**Affiliations:** 1 Division of Epidemiology, University of California, Berkeley, California, United States of America; 2 Institute of Management and Entrepreneurship Development, Dar es Salaam, Tanzania; Vanderbilt University, United States of America

## Abstract

**Background:**

Although women occupy a central position in agriculture in many developing countries, they face numerous constraints to achieving their full potential including unequal access to assets and limited decision-making authority. We explore the intersection of agricultural livelihoods, food and economic security, and women’s sexual and reproductive health in Iringa Region, Tanzania. Our goal was to understand whether the benefits of supporting women in the agricultural sector might also extend to more distal outcomes, including sexual and reproductive health.

**Methods:**

Using the Sustainable Livelihoods Framework to guide data collection, we conducted 13 focus group discussions (FGD) with female (n = 11) and male farmers (n = 2) and 20 in-depth interviews with agricultural extension officers (n = 10) and village agro-dealers (n = 10).

**Results:**

Despite providing the majority of agricultural labor, women have limited control over land and earned income and have little bargaining power. In response to these constraints, women adopt adaptive livelihood strategies, such as alcohol production, that allow them to retain control over income and support their households. However, women’s central role in alcohol production, in concert with the ubiquitous nature of alcohol consumption, places them at risk by enhancing their vulnerability to unsafe or transactional sex. This represents a dangerous confluence of risk for female farmers, in which alcohol plays an important role in income generation and also facilitates high-risk sexual behavior.

**Conclusions:**

Alcohol production and consumption has the potential to both directly and indirectly place women at risk for undesirable sexual and reproductive health outcomes. Group formation, better access to finance, and engaging with agricultural extension officers were identified as potential interventions for supporting women farmers and challenging harmful gender norms. In addition, joint, multi-sectoral approaches from health and agriculture and alternative income-generating strategies for women might better address the complexities of achieving safe and sustainable livelihoods for women in this context.

## Introduction

Agriculture is inextricably linked to poverty, food security, and health in the developing world, and in some regions, women comprise half of the agricultural labor force. [1], [2], [3], [4] However, women face numerous challenges to productive agricultural livelihoods, including unequal access to information, education, inputs (e.g., fertilizer and improved seeds), and markets, lack of capital for investments, fewer assets, restricted decision-making authority over land and earned income, and limited access to agricultural extension services. [1], [3], [5] In addition, land rights, either customary or formal, are dramatically skewed toward men. [6] Supporting women’s central role in agriculture is therefore recognized as a strategy to positively impact agricultural productivity, improve household food security, and decrease vulnerability to shocks, such as illness, drought and price fluctuations. [1].

A growing body of research suggests that the benefits of supporting women in the agricultural sector may also extend to more distal outcomes, including sexual and reproductive health. For example, women’s economic status and autonomy over household decision making are associated with increased use of contraceptives, antenatal care, and skilled birth attendance. [7] Further, economic and food insecurity are positively associated with sexual risk-taking [8], [9] and inversely associated with the dissolution of high-risk relationships and condom use. [9], [10] In addition to these observational studies, emerging experimental evidence supports the hypothesis that economic interventions can improve reproductive health. A social protection program in Mexico (*Oportunidades*), a microfinance and gender and HIV training program in South Africa (Intervention With Microfinance for AIDS and Gender Equity, (IMAGE)), and a cash transfer program for girls in Malawi all found beneficial effects on sexual and reproductive health. [11], [12], [13], [14] In these studies, the authors hypothesized that enabling women with the tools (e.g., skills or capital) to advance economically and/or the agency to define and make choices to benefit from their economic activities (i.e., economic empowerment) would have a positive effect on their behavior. [15].

The causal pathway between economic empowerment and sexual and reproductive health is likely long and complex. Supporting women economically may both directly and indirectly improve health through increased access to health services, better household bargaining positions, and enhanced household and community status, which may increase control over risk behavior and health outcomes (e.g., reductions in transactional sex, gender-based violence). [16], [17], [18] However, there is a paucity of empirical data on these pathways in the rural agricultural setting of Sub-Saharan Africa; this forms the basis of the current study. In order to understand the potential for a livelihood intervention to improve health, we explore the intersection of agricultural livelihoods, food and economic security, and sexual and reproductive health in Tanzania, where women comprise more than half of the agricultural labor force and 85% of adult women in rural areas identify agriculture as their primary occupation. [3], [19] Our goal was to understand whether the benefits of supporting women in the agricultural sector might also extend to more distal outcomes, including sexual and reproductive health.

### Conceptual Frameworks

**Figure 1:**
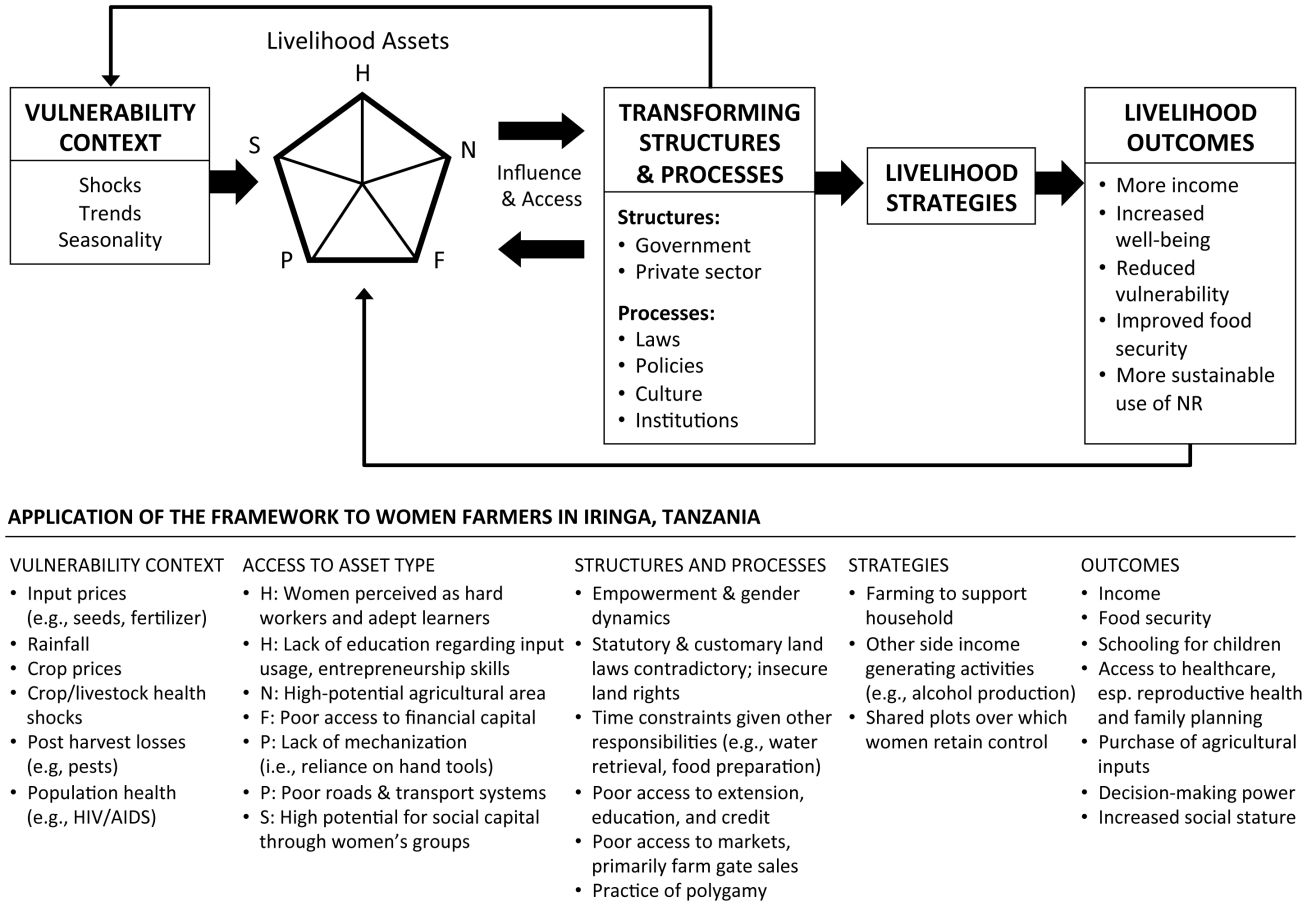
The Sustainable Livelihoods Framework adapted for women farmers in Iringa, Tanzania. Key: H = human capital; N = natural capital; F = financial capital; S = social capital; P = physical capital; NR = natural resources.

The Sustainable Livelihoods Framework (SLF) guided data collection, analysis, and interpretation (Figure 1). [20] The SLF is a people-centered, holistic, asset-based framework for understanding poverty and the livelihoods of the poor. At the core of the livelihoods framework is the *asset pentagon*, which describes the relationship between people’s access to five types of capital: human, social, natural, physical, and financial. These assets combine to produce positive livelihood outcomes. The asset pentagon lies within the *vulnerability context*, which is the external environment over which people have limited or no control (e.g., prices, rainfall). The *transforming structures and processes* are the institutions, policies, and legislation that shape livelihoods. Together, the combination of these factors results in livelihoods strategies that people adopt to achieve their livelihood goals.

Although not the main focus of our study, the high prevalence of HIV/AIDS in Tanzania (5.6% in 2009 [21]) warranted consideration of the gender inequities, social norms, and behaviors that heighten vulnerability to HIV infection and transmission in the general population. For these elements of the study, we were guided by the proximate-determinants conceptual framework for HIV/AIDS, which posits that the sociocultural and economic context influences the proximate behavioral determinants of HIV exposure, such as new partner acquisition and condom use, which directly affect the likelihood of HIV transmission. [22] We also referenced an adaptation of the SLF which shows how HIV/AIDS affects, and is affected by, livelihoods. [23].

## Methods

The study was conducted as part of the impact evaluation of a large agricultural subsidy program in Tanzania, the National Agricultural Input Voucher Scheme. The subsidy program, launched in 2009 by the Tanzanian Ministry of Agriculture, Food Security and Cooperatives, aims to increase household incomes and bolster food security by distributing vouchers for a 50% subsidy on a package of inputs (fertilizers and improved seeds) to 2.5 million maize and rice farmers. There is a specific emphasis on targeting female-headed households for the program. The ongoing impact evaluation will evaluate the program’s effects on input use, productivity (yield per hectare), profitability, and household welfare.

We complemented the quantitative impact evaluation with a qualitative study to better understand the potential for agricultural interventions to affect women’s economic empowerment and sexual and reproductive health. Qualitative methods were identified as the most appropriate approach because we sought to understand the socio-cultural context of women living in subsistence farming households in rural Tanzania and the potential pathways between economic empowerment and sexual and reproductive health. [24] Although the subsidy program was the underlying motivation for the study, we examined a broad spectrum of issues; this report focuses on the findings related to economic empowerment and sexual and reproductive health.

The study consisted of 13 focus group discussions (FGD) with female (n = 11 FGDs) and male farmers (n = 2 FGDs) who were purposefully selected to represent groups of interest, including: married heads of household, unmarried or widowed heads of household, and beneficiaries and non-beneficiaries of the agricultural subsidy program (Table 1). Two of these groups were limited to women living with HIV/AIDS. In addition, given that agricultural extension officers and village agro-dealers are key actors in the local agricultural economy and may frequently interact with women farmers, we included these groups in the study population. Agriculture extension officers are government employees (male and female) who serve multiple villages and transfer agricultural technology, knowledge, and advice to rural farmers. Agro-dealers are village-based businessmen and women who are the main conduit of agricultural supplies (e.g., fertilizer, seeds, and pesticides) and advice to subsistence farmers. We conducted 10 in-depth interviews with extension officers and 10 in-depth interviews with village agro-dealers, who could be male or female. In-depth interviews were conducted with extension officers and agro-dealers because they are dispersed throughout the region and assembling FGDs would have been impossible.

**Table 1:** Description of focus group discussion (FGD) participants in Iringa Region, Tanzania.

Group	No. of FGDs	Total participants	Inclusion criteria	Age (median, range)
Widows	4	33	• Head of household	43 (27–70)
			• Female	
			• ≥18 years of age	
Women living with HIV/AIDS	2	17	• Head of household	35 (25–62)
			• Female	
			• ≥18 years of age	
Married women	2	17	• Head of household	30 (24–47)
			• Female	
			• ≥15 years of age	
Unmarried women serving as heads of household	3	25	• Head of household	26 (20–37)
			• Female	
			• ≥15 years of age	
Male farmers	2	17	• Head of household	42 (24–71)
			• Male	
			• ≥18 years of age	

Agriculture was the primary income-generating activity for all participants.

### Study Setting and Population

Our study was conducted in the Iringa Region of Tanzania in July-September 2011. Iringa is an agriculturally high potential area and is known as one of the “Big Four” regions in terms of staple food production. Maize is the most common crop in addition to potatoes, rice, beans, cassava, tea, tomatoes and sunflower seeds. Sixty-nine percent of the adult female labor force in Iringa works in agriculture, [19] and Iringa has the highest HIV prevalence in the country, with 16.8% of adult women found to be HIV positive in a 2008 survey. [25].

FGDs participants met the following inclusion criteria: 1) head of household or married to the head of household; 2) agriculture as the main income generating activity (primarily subsistence farming); 3) already planted or planning to plant crops in the current season in the Iringa Region; and 4) at least 18 years of age or at least 15 years of age in the case of female heads of household. In-depth interviews with agricultural extension officers and village agro-dealers required that participants serve the selected village in either of these roles and were at least 18 years of age.

### Recruitment

Two of the five districts in Iringa Region where rollout of the subsidy program was underway were randomly selected (Mufindi and Kilolo), and within each district, we randomly selected four villages as study sites. A letter of introduction was sent from the Ministry of Agriculture, Food Security and Cooperatives to the District Agricultural and Livestock Development Officer in Kilolo and Mufindi, and the research team met with the Regional Medical Officer and health officers in both districts at study initiation. At each village, the three-person local research team (female facilitator, female note-taker, and male interviewer) first met with the Village Executive Officer to discuss the study, identify possible FGD participants from village rosters, and for introductions to the village’s agro-dealer and extension officer. Most FGD participants were purposefully recruited from existing women’s groups (e.g., HIV/AIDS support groups) or through participant referral. Potential participants were informed verbally about the purpose and nature of the study and if interested, they were invited to the discussion, which was typically held the next day.

### Data Collection

Most data collection activities were conducted in Kiswahili by three local research staff trained in qualitative methods (in some cases, local languages were used with the assistance of an interpreter). A semi-structured interview guide approach was utilized whereby pre-determined issues are covered (to ensure systematic data collection) but the interviewer is free to change the sequence and the wording of questions during the course of the discussion or interview. [26] Nine of twelve FGDs and 19 of 20 individual interviews were digitally recorded with consent from participants (two FGDs and one individual interview were not recorded at participant request and one FGD was not recorded due to recorder malfunction). In these cases, the moderator and note-taker’s detailed notes were used to reconstruct overall themes and specific quotes.

*FGDs:* FGDs had 8–9 participants and were conducted by the female facilitator and female note-taker. [26] After initial introductions, the facilitator explained the study and obtained written, informed consent (or a mark/thumbprint indicating consent). FGDs lasted approximately 1–2 hours and typically took place in a private space (e.g., a village meeting room or outside). Either before or after the discussion, participants completed a brief survey on basic demographic characteristics. Participants were compensated with a kanga (a piece of colorful printed fabric worn by women, approximate value USD$4) or cell phone airtime.

Given issues of confidentiality and the sensitivity of the subject matter, FGD participants were encouraged to discuss group- rather than individual-level experiences and perceptions. The discussion guide, developed from the conceptual frameworks, was comprised of three main types of questions. Main questions addressed the research themes, and were broad in nature (e.g., “Are there special challenges that women farmers face?”). Follow-up questions and probes encouraged deeper, more detailed information (e.g., “Do women heads of household get to make the final decisions about land, such as what crops to grow, and whether to sell the land? What about married women?”) [26]. There were 8 to 9 main questions in each FGD, each of which had 2 to 4 open-ended follow-up questions or probes. Although the FGD guide included a broad spectrum of issues, the data presented here are from findings related to three main concepts: 1) women’s empowerment and/or inequity in agriculture, 2) food security, and 3) sexual and reproductive health, including factors that heighten the risk of HIV/AIDS. Throughout each FGD, the note taker recorded common responses, any new or unexpected ideas, group agreement/disagreement, outlying responses, and non-verbal cues. At the completion of each discussion, interview summaries were created by the research team to record first impressions and suggested improvements for future discussions.

*In-depth interviews:* Individual interviews were one-on-one interviews conducted by a male interviewer with agricultural extension officers and village agro-dealers. Using a similar interview guide approach, the interviews focused on regional agricultural practices and women’s empowerment and/or inequity in agriculture. A special emphasis was placed, within each of these topics, on the participant’s interaction with women. For example, extension officers were asked if women sought him/her for advice and whether special efforts are made to reach women farmers.

### Analysis

Audio files were translated and transcribed verbatim into English by two of the local research staff. Two members of the study team (SM & LR) then independently read and coded a subset of six transcripts to begin identifying and coding organizational and substantive themes within the data. Organizational themes were defined as those that responded directly to the main, *a priori* research questions. Substantive themes were defined as those that were not anticipated, either in their content or specificity, by the study team based on our conceptual framework or discussion guide, but emerged from the data. [24] For example, although we anticipated that women might engage in side businesses or other income-generating activities, the universal nature of alcohol production as women’s primary income generating activity was not anticipated and resulted in a new substantive theme (described later). After double-coding of the initial six transcripts to establish a preliminary list of organizational and substantive themes, the remaining transcripts were then divided between the two coders and reviewed for further elaboration and clarification of the themes. The two coders met weekly throughout the coding process to resolve coding or interpretation differences through discussion and debate. When differences emerged that could not be resolved, when additional context was needed, or when the two coders felt that additional discussion was needed on a particular theme or concept, a member of the field team (WW) was brought into the discussion. Once consensus was reached on a final list of themes, transcripts were revisited for additional dimensions, including whether the responses represented individual experiences or group-level norms, the degree to which the ideas represented factual or perceived experiences, and the extent to which themes were salient only for certain subgroups of participants. [26] We compared our findings back to the conceptual frameworks to determine how the data inform, substantiate, or deviate from previous models.

### Human Subjects Protection

All participants in the study provided written, informed consent (or a mark/thumbprint indicating consent). This study and consent procedures were approved by the National Institute for Medical Research in Tanzania and the University of California, Berkeley Committee for Protection of Human Subjects.

## Results

Thirteen focus groups were held; eleven with women farmers and two with male farmers (Table 1). On average, focus group discussions had 8 to 9 participants and lasted 67 minutes (range 42 to 97 minutes). Ten in-depth interviews each were conducted with agro-dealers (3 female, 7 male) and agricultural extension officers (2 female, 8 male). There was only one direct refusal for an in-depth interview. We present the results relating to the following concepts: 1) women’s access to and use of livelihood assets, including constraints to productive agricultural livelihoods, 2) adaptive livelihood strategies, including alcohol production, and 3) the convergence of risk facilitated by women’s livelihood strategies. The specific organizational and substantive themes related to these concepts are summarized in Table 2.

**Table 2:** Description of organizational and substantive themes from focus group and in-depth interview participants in Iringa Region, Tanzania.

Organizational	Substantive
• Women farmers have high productive potential.	• Economic instability motivates frequent adoption of adaptive livelihood strategies by women farmers.
• Women face concurrent and often competing responsibilities foragricultural productivity and supporting the household.	• Subtheme: Alcohol production serves as the most frequent and logical adaptive livelihood strategy for women farmers given other agricultural and household responsibilities.
• Women face numerous and diverse constraints to productive agricultural livelihoods, including inadequate land ownership, lack of control over assetsor income, and competing health demands.	• Alcohol consumption is widespread among both men and women and often facilitates sexual risky behavior.
• Gendered agricultural practices reinforce women farmers’ disadvantage.	• Economic instability directly and indirectly enhances women’s sexual risk.

### Women’s Access to and use of Livelihood Assets

Participants in both FGDs and individual interviews confirmed the integral role of women in agricultural production and ensuring household food security. When asked to describe what it is like to be a female farmer, nearly all respondents described farm labor, particularly for subsistence crops, exclusively as a women’s responsibility. This “productive” responsibility came in concert with myriad other family and social responsibilities, [1] including child caregiving, food preparation, water retrieval, household maintenance, caring for sick family members, and social interactions to build community.

Participants described women as having access to a diverse array of livelihood assets, including the region’s natural resources (e.g., favorable soil and weather conditions), their own skills, knowledge, and abilities (i.e., human capital), and social resources (e.g., groups) to pursue their livelihood objectives. Women were described by men (in both FGDs and individual interviews) as hard workers, “*eager to learn*”, and highly capable of running a farm. A male extension officer noted that “*educating 1 woman is like educating 100 or 200 men*.” Access to groups was cited as an asset, enabling women to share knowledge and labor (as a means to respond to illness), provide social support and encouragement, and access financing (when used as rotating savings and credit associations (ROSCAs)).

However, despite their central position in agricultural production, participants universally described women’s striking lack of autonomy and decision-making authority over land use and earned income, combined with a lack of secure land rights, when asked to discuss special challenges faced by women farmers. Across focus groups and interviews, participants agreed that men controlled earned income from agriculture, regardless of the extent of women’s participation in agricultural production or her role in the household. These narratives largely described group-level experiences, although the few individual experiences described did not deviate from these shared general perceptions:

“*A husband can allow you to use his farm but later on he snatches it from you…with your crops.*” – Widow

“*[Women’s] life is real hard, they take all household responsibilities and productive activities…women have no say in the income though they are the main producers in the household.*” – Male agro-dealer

Responses varied when participants were asked to describe the extent to which women (particularly widows) could own land and the context of ownership. The most commonly expressed sentiment, particularly by female focus group participants, is captured in the following female participant’s response to a question about who controls the farms after a husband’s death: “*They are taken by the relatives and the children, for his wife it is gone forever.”* In slight contrast, interview and male focus group participants acknowledged that women could own and inherit land, but that land division was often inequitable and justified as a way to prevent “developing” families outside of the clan. A male FGD participant noted that *“for the inherited farms, the land is divided with equal rights but the woman will get a small farm, a man will get more…they consider that a women is married therefore can’t take the farm to the kids of another clan.”* One married woman highlighted the precarious situation this places women in who wish to marry: *“For some families, they forbid you to get married unless you leave everything that belonged to your late husband.”* However, a handful of FGD participants agreed that despite being inequitable in the past, women’s ability to own or manage small parcels of land following the death of her husband was improving in recent years. One widow explained that the *“behavior of relatives snatching everything from the widow is now diminishing.”* These varied responses may reflect differences in customary law between villages or contradictions between statutory and customary law. [27].

In addition to insecure land rights, women were described as having poor access to agricultural education and both agro-dealers and extension officers noted that women were often lacking basic entrepreneurial skills, such as how to properly use inputs or compute profits. In addition, women’s poor access to financial and physical capital results in a reliance on hand tools, including hand hoes and handcarts, which is particularly challenging given that farms are often located far from the household.

Especially when combined with their other responsibilities, it was apparent that any shocks or disruptions could have a tremendous cost for women and their families. For example, with regard to the impact of pregnancy on agricultural productivity, one agro-dealer explained, “*there is no family planning thus women lose their working capacity when still young.*” A woman living with HIV infection explained the labor impact of clinical care and antiretroviral therapy: *“*S*omeone might go to town and stop all that she had to do that day, for the whole day she is in the town taking medication.”* Thus, sexual and reproductive health demands were described as competing with income-generating activities.

### Women’s Adaptive Livelihood Strategies

In all but two of the female FGDs, participants noted that women frequently adopt other income-generating activities (e.g., owning a small kiosk or store, selling snacks) on top of farming responsibilities, recognizing that they cannot rely on their husbands’ earnings for important household expenses. A widow emphasized that *“what you care is that my family can get the food and does not suffer and that's it. You cannot count on his money.”* For some subgroups (e.g., widows or women living with HIV infection), side businesses were viewed as essential. A widow explained, *“she [HIV positive woman] has to get an education and a loan to enable her to do small scale businesses to help her for there is no other alternative.”* However, the extent of women’s engagement in other income-generating activities was often constrained by the time required for other household responsibilities.

In two-thirds of FGDs, participants reluctantly acknowledged that some women in their communities exchanged sex to procure money, food, or other goods to alleviate their economic insecurity. One widow explained how this might happen, and the potential for unintended consequences: *“A child is starving … someone gives me five thousand, I go with him in order to get some needs of the child, but later the calamity starts.”* Another unmarried woman used an analogy of agriculture to describe transactional sex: “*that’s her hoe, what else can she use*?” Some women hesitated to directly attribute transactional sex to economic and food insecurity; rather, they connected economic insecurity to joining a family headed by a man with a good income:

“*Few women have sex for money or food. For most, they better get married [as] a second wife to the man who has big farms and animals, than doing sex for food.*” – Widow

This nuanced viewpoint highlights the subtle difference between transactional sex to obtain essential items versus engaging in a relationship with the potential for longer-term economic stability.

Brewing wine or beer (typically *ulanzi*, fermented bamboo sap, or *komoni*, a maize-based local beer), or selling maize to others who would use it to brew alcohol, was by far the most common income-generating activity reported by women. Unlike other potential businesses, women explained that little or no capital was required as an up-front investment and it could be done using crops (e.g., maize) they are already growing. A female FGD participant noted, *“if you don’t have any other businesses to do then you will only make local brew.”* This income was described as largely under women’s control and typically used to support the household, including school, food and medical costs and informal payment to farm laborers. As one female focus group participant explained: *“Many men nowadays allow their wives to have their private projects but you have to take care of the family*.*”* This highlights the assumption that income generated from women’s adaptive livelihood strategies be used to support the household; the same is not true for money generated by men’s activities. A male extension officer and a married woman both echoed this sentiment:

“*Women do involve in preparing and selling alcohol so that they can get income to run their household, taking children to school, hospital, find food, etcetera, as their husband does not contribute any to the households and they will always take money from their wives.*”

“*The hardship comes in taking the kids to school, I pay for two children in secondary school, I also have three in primary school, one in nursery school, if I don’t sell local brew and do farming I cannot keep up my family.*” – Married woman

In this way, making local brew or selling crops for brewing filled a critical gap to meet a household’s food, medical, and educational needs.

### A Convergence of Risk: Risk Environments Enhanced by Adaptive Livelihood Strategies

In response to a lack of control over their earnings, weak bargaining power, and heavy burden of domestic tasks, participants described how women adopted livelihood strategies that directly or indirectly enhanced their risk for undesirable health outcomes. Exchanging sex for money or food clearly increased women’s risk of sexually transmitted infections, including HIV, although this was generally described as rare. However, focus groups revealed a direct connection between women’s most common income generating activity and their risk for adverse health consequences. As described above, brewing alcohol was a logical strategy that women adopted in order to earn extra income given their time and capital constraints. FGD participants described a high demand for alcohol in their communities, and women’s role in brewing alcohol often brought them into bars and other social settings where alcohol consumption was common. As one participant described “*The main activity after farming, house work, and picking of vegetables is selling the local brew in the bars and chatting with friends*.” In one community, alcohol consumption was pervasive enough that a curfew was established for women at 20∶00 so that they would leave the bars and return home (no curfew existed for men).

Alcohol consumption was also described as contributing to the economic instability of families, and related to women’s lack of control over earned income. For example, one widow explained, *“He uses every cent you earn from your farms to drink.”* An agro-dealer noted, “*women and their husband[s] have different farms, but income from [the] husband’s farm are just for alcohol drinking*.” Further, in a majority of FGDs, participants named alcohol consumption as the primary driver of risky sexual behavior in their communities for both men and women:

“*The spreading of HIV/AIDS is very high in this area because many people are engaging carelessly in sexual intercourse. This situation is caused by drinking local beer, for when a person been drunk he becomes very careless.*” – Widow

“*One day you can decide to go and mix yourself in a local club, you meet your friends… Like women, they do go there without any money but on arrival men buy them a drink…they find themselves drinking and lately their senses shifts. They advise each other and run together and that’s it.*” – Male FGD participant

“*Local brew drinking disturbs a lot, especially in February, March and April. These are the months for Ulanzi. You take water [local brew] till you are out of your senses, you get HIV, then you are already gone.*” – Male FGD participant

“*When you are drinking together and a man orders a seller to give a litre to one woman among you, the rest have to finish the litre and quickly leave. They should leave a space for a man and a woman as she will surely pay for that litre.*” – Widow

Together, the narratives delineate the opposing effects of alcohol on women’s economic security and health. On one hand, alcohol production is an important income-generating activity for women over which they maintain control and use to invest in the household, including children’s education. Conversely, alcohol consumption was associated with higher-risk sexual behavior that could potentially have deleterious consequences on their sexual and reproductive health.

## Discussion

In this study in Iringa Region of Tanzania, we found that despite women providing the majority of agricultural labor, they have little authority over earned income and have poor access to and control over assets, including land. This has been documented in other studies and is the underlying motivation for efforts by the World Bank, the International Food Policy Research Institute (IFPRI), the Food and Agriculture Organization of the United Nations (FAO), and other agencies to support women working in the agricultural sector and close the gap in women’s access to agricultural education, technology, land, markets, and financial capital. [1], [3], [28], [29] In addition, we found that most women farmers adopt additional livelihood strategies as a rational response to their inequitable access to agricultural assets and lack of authority over earned income. An unexpected finding of our study was that the most common income-generating activity described was unquestionably alcohol production, which has the dual potential to both bolster incomes and heighten vulnerability to poor sexual and reproductive health outcomes.

Women’s central role in agriculture facilitated their engagement in alcohol production, which was sold or used as in-kind payment to farm laborers. In contrast to agricultural earnings, women appeared to have decision-making authority over money earned from alcohol production, with the expectation that it be used to invest in the household. Given that alcohol production used existing assets (e.g., maize) and required a minimal time investment, it fit within the time limitations of women’s heavy burden of domestic responsibilities. In this way, alcohol production was a natural economic activity for women to adopt. This finding validates one portion of the SLF in that women identify ways to capitalize on available assets to succeed economically and to *benefit* from these economic activities by retaining some control over income – all within the constraints of the structures and processes that shape agricultural livelihoods (Figure 1). Our study found that these constraints were formidable and prevented women from engaging in other livelihood activities.

The finding that alcohol production is the most common income-generating activity adopted by female farmers is not new. The manufacture of local brew for cash sale, rather than trade or barter, has been under the purview of women in eastern Africa at least since the 1930’s. [30] Women have typically functioned as producers and men as consumers, thus redistributing wealth from men to women. [30], [31] In our study, the gendered roles of production and consumption were not so clearly delineated, as women also reported drinking frequently themselves. [30], [31] Brewing has been called “*the single most significant economic activity for rural women*” in Sub-Saharan Africa, one that “*provides higher levels of income than any other business or employment*”. [31] In one region of Uganda, beer is known as the “cattle of women”, referring to its social and economic value. [32] A anthropological study based in this area found that beer and beer dregs represented an important source of energy in the household in its own right, and women’s earnings from selling beer on the retail market were often spent on domestic essentials and nutrient-dense foods for the household (e.g., beans, fish, greens, nuts and seeds, and tomatoes). [32] Further, households of women who brewed and sold their own beer had higher average energy and nutrient intake than households where women did not participate in brewing. [32] This finding suggests the possibility of a direct link between brewing and nutrition in the rural setting, as alcohol production is a major source of income for women who are responsible for their children’s nutrition. Notably, none of our participants described purchasing or trading for food with the income earned from brewing; however, given that this was not an *a priori* focus of our research, we may not have probed in enough detail to elicit these types of responses.

The benefits of brewing as a livelihood strategy for women and their households are countered by the significant social and health consequences of alcohol misuse. For example, an unexpected finding of our study was the potential ways in which women’s central role in alcohol production, in concert with the ubiquitous nature of alcohol consumption, placed women at risk by enhancing their vulnerability to unsafe or transactional sex. Alcohol is widely recognized as a driver of behaviors that facilitate the likelihood of transmission and acquisition of STIs, including HIV, and unplanned pregnancy in Sub-Saharan Africa. [33], [34] For example, multiple studies have reported the association between “impaired sex” and a higher likelihood of unprotected sex, multiple sexual partners, and transactional sex. [33], [34], [35] This has been well documented in the African setting [36], [37], including the use of alcohol as currency for sexual exchange [38], which was also reported in our study. Further, integration of alcohol-reduction interventions into primary care for HIV infected outpatients is currently being explored as a strategy to reduce sexual risk behaviors and improve clinical outcomes. [39], [40].

In our study, participants acknowledged that women’s risk of unsafe sex increased as a result of alcohol use both directly through their own behaviors and indirectly through their partner’s behaviors – thus alcohol use is an “underlying determinant” (in the proximate-determinants model) that shapes the patterns of partnership formation and sexual risk behavior in a community. [22] These behaviors may increase the size and linkages of sexual networks that could facilitate the spread of STIs, including HIV. [41] To our knowledge, our study is the first to describe this dangerous confluence of risk for female farmers: in this part of Tanzania, women supply much of the community’s alcohol as an adaptive and essential livelihood strategy and must simultaneously contend with the consequences of risky sex resulting from alcohol use.

Alcohol use is also associated with a host of negative health and social consequences other than HIV, STIs, and unintended pregnancy, including alcohol dependency, liver disease, cardiovascular disease, fetal alcohol syndrome, violence, and unintentional injury such as road traffic accidents. [42] In 2010, the World Health Assembly issued a draft global strategy to reduce the harmful use of alcohol, which emphasized the importance of a legal framework to reduce the physical availability of alcohol that encompasses restrictions on both the sale and serving of alcohol. [43] Indeed, some countries in Sub-Saharan African have recently bolstered their regulatory policy, including South Africa, Botswana, Zambia and Kenya, who have considered or passed legislation to limit alcohol manufacture, consumption, and/or advertising. [34], [44] Although Tanzania’s alcohol policy includes excise taxes on alcohol, a minimum drinking age of 18, a zero tolerance policy for drinking and driving among young drivers, regulations on alcohol advertising and sponsorships, and restrictions for on- and off-premise sales of alcoholic beverages, [45] enforcement of such policies may be difficult in the rural setting, especially for home brews. Further, higher taxes on commercial alcohol may have the unintended consequence of driving consumption away from the commercial market toward the home brew market, which is not regulated for quality and may include dangerous additives to increase potency. [44], [46] In rural settings, effective strategies may include community mobilization to raise awareness and change social norms around drinking, changing drinking settings (e.g., integration of HIV prevention activities with drinking venues) and patterns of consumption (i.e., binge drinking), and educational interventions within schools. [33], [34] However, without careful consideration of the reality of alcohol’s dual role for women, increased regulation and reducing the demand for alcohol may run the risk of addressing one issue (alcohol use and its consequences) at the expense of another (increasing women’s economic dependency on men).

A goal of our study was to explore the pathways through which economically empowering women farmers may improve sexual and reproductive health. In this regard, we found that current conceptual models poorly described the complexity of the social and economic environment in this region of Tanzania. For example, the SLF does not adequately capture interactions between some dimensions of the model, such as the ways in which livelihood strategies like alcohol production can negatively influence the “vulnerability context” (i.e., through the spread of HIV or STIs or supplying a community’s alcohol) or the ways through which these strategies perpetuate imbalanced power relations (i.e., “transforming structures and processes”) if women do not challenge social norms. For example, in our study, despite recognizing gender inequity, there were no attempts by study participants to challenge the status quo, other than short-term coping strategies, such as group formation or the adoption of additional, small-scale, income-generating activities. Likewise, although the proximate-determinants model for HIV/AIDS acknowledges “socioeconomics” and “sociocultural” factors as underlying determinants, it does not sufficiently capture a more nuanced and perhaps cyclical system in which livelihood strategies can be both beneficial for some health outcomes and at the same time potentially reinforce harmful behaviors that increase the likelihood of other poor health outcomes.

Our data suggest several possible intervention points to enhance women’s empowerment in agriculture and potentially improve sexual and reproductive health. Group formation was frequently noted as a way to share information and advice and facilitate interactions with overstretched extension officers. However, groups alone are unlikely to address the underlying social determinants of women’s health and be “gender transformative”. [47] Access to finance or capital also emerged as an intervention point; women in our study often deferred to alcohol production because of a paucity of other opportunities. There is some evidence that financial programs can achieve laudable health effects without the unintended consequences on risk behavior. For example, the IMAGE study in South Africa combined a women’s microfinance program with a gender and HIV training curriculum. [13] The program reduced partner violence by 55% and, among younger loan recipients, the program increased household communication about sexual health, HIV testing, and protected sex with casual partners. [48] Similarly, *Oportunidades*, a Mexican cash transfer program, increases the use of contraception [11], [49], [50], [51], [52], prenatal care (beyond program requirements) [50], [53], skilled birth attendance [50], [53], and women’s autonomy and household bargaining power [12], [49] – positive “spillover effects” that are not directly linked to the conditions associated with the cash transfers. [54], [55].

However, programs directly related to agriculture may be the most salient in this context. Extension officers in particular have the potential to be powerful agents of change. Their narratives suggest that they recognize gender inequities; however, like the women in the study, there was typically no action taken beyond passive recognition of gender inequities and harmful social norms. It is possible that increasing extension officers’ sensitization to gender issues and enabling them with tools to challenge gender norms could be transformative. Extension officers could not only advocate for the adoption of technology – including fertilizers, improved seeds, and labor saving physical assets – but could spread information about land rights and make linkages to legal resources, could facilitate the adoption of higher-value crops, and connect solitary women together into groups to build social capital and enhance access to finance. They could also play an important role by engaging with men to achieve a truly “gender synchronized” approach to transforming social norms. [47] Finally, short-term subsidy programs, like the ongoing subsidy program in Tanzania, are designed to “kick-start” production so that program graduates have saved enough (after several years of program participation) to independently purchase inputs on the market.

It is difficult to disentangle the myriad gender-related obstacles faced by women in the region from the ubiquitous risk and consequences of the HIV epidemic. This is particularly true in a region like Iringa where up to one in six adult women is living with HIV infection. [25] In rural settings, women’s success in agriculture partially depends on her success in avoiding HIV infection, as HIV/AIDS-related stresses (such as time, medical costs, and physical effects of the disease) impact her ability to devote time and labor to agriculture; indeed, the HIV epidemic has had devastating effects on agricultural productivity. [56], [57], [58]. But the reverse is also true; ensuring women’s empowerment in agriculture can reduce higher-risk behaviors (e.g., transactional sex) that may heighten vulnerability to HIV infection. [8], [9], [59], [60] In addition to food insecurity and poverty, insecure land rights further diminish women’s economic security and erode their sexual autonomy and bargaining power. [61], [62] In this way, HIV/AIDS both affects and is affected by women’s empowerment in the agricultural sector. [23] In our study, women’s inconsistent understanding of their often contradictory land rights and the competing agricultural and medical demands faced by women living with HIV lends support to these postulated relationships. [23], [62].

Our study has important limitations and strengths that must be considered when evaluating the findings. Our goal was to collect in-depth data from a small group of individuals to describe the relationships between women’s empowerment in agriculture and health. However, our data may not be generalizable to all women working in the agricultural sector, or to other regions in Tanzania that may experience different levels of productivity and food security and have different social and cultural norms. We placed particular emphasis on female heads of household who worked primarily on their own family farm. In contrast, women who work as casual wage laborers without a farm may represent the poorest and most disenfranchised women in a community and may have different perspectives on economic empowerment than the women in our study. We also selected women for the study using peer-recruitment strategies, including existing women’s groups, which may partially explain the discussions about the benefits of group formation. Finally, individual interviews with women were not conducted; as a result, the narratives largely reflect social norms and expectations rather than individual-level experiences. Future work could focus on women’s individual experiences and how they corroborate or deviate from the conceptual frameworks and community norms. Our study also has significant strengths, including a large number of focus groups with women and men, the inclusion of HIV-infected women, as well as triangulation of data with agro-dealers and extension officers.

### Conclusion

The agricultural sector could play a significant role in poverty reduction in developing countries and is critical for achieving the Millennium Development Goals. [63] The value of women’s empowerment in agriculture is therefore unequivocal. As an important source of agricultural labor and food production in many rural settings, women are well positioned to have a transformative role in agricultural growth and economic development. Further, economically empowered women have more autonomy over their lives, including increased household bargaining power and social stature, access to health services, decision-making authority over childbearing, and their children have better educational and health outcomes, interrupting the devastating cycle of poverty and poor health. [64] However, in the absence of control over earnings generated from agriculture, often coupled with the inability to rely on income earned by male partners or family members, women in our study adapted the resources and skills readily available to them to generate income that remains under their control. In this case, alcohol production may help them achieve their livelihood goals but may also increase the likelihood of deleterious sexual and reproductive health outcomes, as well as other poor health and social outcomes. Better integration of supportive systems for women – from agricultural education to family planning – as well as joint, multi-sectoral approaches from health and agriculture, would better address the complexities of achieving safe and sustainable livelihoods in this context. In addition, alternative livelihood strategies for women that have the same income-earning potential as brewing may protect women’s (limited) autonomy over earned income and help to reduce the ubiquitous availability of alcohol. Finally, regulatory policy and changes in community social norms around alcohol consumption may reduce the harms associated with alcohol use.

## References

[1] The World Bank (2009) Gender in Agriculture Sourcebook. Washington, D.C.

[2] Food and Agriculture Organization (1997) Women and sustainable food security. Rome: FAO.

[3] Food and Agriculture Organization (FAO) (2011) Women in Agriculture: Closing the gender gap for development. Rome: United Nations.

[4] The World Bank (2007) World Development Report 2008: Agriculture for Development. Washington, D.C.

[5] Quisumbing A, Pandolfelli L (2008) Promising Approaches to Address the Needs of Poor Female Farmers. Washington, D.C.: International Food Policy Research Institute.

[6] Rural Development Institute (2009) Annual Report. Secure Land Rights: The Key To Building A Better, Safer World. Seattle, WA.

[7] Ahmed S, Creanga AA, Gillespie DG, Tsui AO (2010) Economic status, education and empowerment: implications for maternal health service utilization in developing countries. PloS one 5: e11190. 20585646 10.1371/journal.pone.0011190 PMC2890410

[8] Weiser SD, Leiter K, Bangsberg DR, Butler LM, Percy-de Korte F, et al.. (2007) Food insufficiency is associated with high-risk sexual behavior among women in Botswana and Swaziland. PLoS Med 4: 1589–1597; discussion 1598. 10.1371/journal.pmed.0040260 PMC2039764 17958460

[9] Miller CL, Bangsberg DR, Tuller DM, Senkungu J, Kawuma A, et al (2011) Food Insecurity and Sexual Risk in an HIV Endemic Community in Uganda. AIDS Behav 7: 1512–9. 10.1007/s10461-010-9693-0 PMC3110536 20405316

[10] Greig FE, Koopman C (2003) Multilevel analysis of women’s empowerment and HIV prevention: Quantitative Survey Results from a preliminary study in Botswana. AIDS Behav 7: 195–208. 14586204 10.1023/a:1023954526639

[11] Ladmadrid-Figueroa H, Angeles G, Mroz T, Urquieta-Salomon J, Hernandez-Prado B, et al (2010) Heterogeneous impacts of the social programme Oportunidades on use of contraceptive methods by young adult women living in rural areas. Journal of Development Effectiveness 2: 74–86.

[12] Adato M, de la Briere B, Mindek D, Quisumbing A (2000) The Impact of Progresa on Women’s Status and Intrahousehold Relations. Washington, D.C.: International Food Policy Research Institute.

[13] Pronyk PM, Hargreaves JR, Kim JC, Morison LA, Phetla G, et al (2006) Effect of a structural intervention for the prevention of intimate-partner violence and HIV in rural South Africa: a cluster randomised trial. Lancet 368: 1973–1983. 17141704 10.1016/S0140-6736(06)69744-4

[14] Baird SJ, Garfein RS, McIntosh CT, Ozler B (2012) Impact of a cash transfer program for schooling on prevalence of HIV and HSV-2 in Malawi: a cluster randomized trial. Lancet. 10.1016/S0140-6736(11)61709-1 22341825

[15] Golla AM, Malhotra A, Nanda P, Mehra R (2011) Understanding and Measuring Women’s Economic Empowerment. Definition, Framework and Indicators. Washington, D.C.: International Center for Research on Women (ICRW).

[16] United Nations Population Fund (2007) Women’s Economic Empowerment: Meeting the Needs of Impoverished Women. New York.

[17] Kim J, Pronyk P, Barnett T, Watts C (2008) Exploring the role of economic empowerment in HIV prevention. Aids 22 Suppl 4S57–71. 10.1097/01.aids.0000341777.78876.40 19033756

[18] Levine R, Lloyd CB, Greene M, Grown C (2008) Girls Count: A Global Investment & Action Agenda. Washington, D.C.: Center for Global Development, Population Council, & International Center for Research on Women.

[19] National Bureau of Statistics (NBS) [Tanzania] and ICF Macro (2011) Tanzania Demographic and Health Survey 2010. Dar es Salaam, Tanzania: NBS and ICF Macro.

[20] Department for International Development (1999) Sustainable Livelihoods Guidance Sheets. London.

[21] UNAIDS (2010) Report on the Global AIDS Epidemic. Geneva.

[22] Boerma JT, Weir SS (2005) Integrating demographic and epidemiological approaches to research on HIV/AIDS: the proximate-determinants framework. J Infect Dis 191 Suppl 1S61–67. 15627232 10.1086/425282

[23] Gillespie S, Kadiyala S (2005) HIV/AIDS and Food and Nutrition Security: From Evidence to Action. Washington, D.C.: International Food Policy Research Institute.

[24] Maxwell JA (2005) Qualitative Research Design: An Interactive Approach. Thousand Oaks, California: Sage Publications, Inc.

[25] National Bureau of Statistics (NBS) (2007–2008) Tanzania HIV/AIDS and Malaria Indicator Survey: Preliminary Report. Dar es Salaam.

[26] Ulin P, Robinson E, Tolley E (2005) Qualitative Methods in Public Health: A Field Guide for Applied Research. San Francisco, CA: Jossey-Bass. 1–318 p.

[27] Food and Agriculture Organization of the United Nations (2010) Gender and Land Rights Database: United Republic of Tanzania.

[28] International Food Policy Research Institute (IFPRI) (2012) Women’s Empowerment in Agriculture Index. Washington, D.C.

[29] Food and Agriculture Organization (FAO) (2011) Women and Agriculture: Closing the Gender Gap For Development. Rome.

[30] Willis J (2002) Potent Brews: A Social History of Alcohol in East Africa, 1850–1999. Oxford: James Currey. 320 p.

[31] McCall M (1996) Rural brewing, exclusion, and development policy-making. Gend Dev 4: 29–38. 12347713 10.1080/741922167

[32] Dancause KN, Akol HA, Gray SJ (2010) Beer is the cattle of women: sorghum beer commercialization and dietary intake of agropastoral families in Karamoja, Uganda. Social science & medicine 70: 1123–1130. 20117870 10.1016/j.socscimed.2009.12.008

[33] Chersich MF, Rees HV (2010) Causal links between binge drinking patterns, unsafe sex and HIV in South Africa: its time to intervene. International journal of STD & AIDS 21: 2–7. 20029060 10.1258/ijsa.2000.009432

[34] Fritz K (2011) Alcohol and Risky Sex: Breaking the Link. Washington, D.C.: USAID.

[35] Browne FA, Wechsberg WM (2010) The intersecting risks of substance use and HIV risk among substance-using South African men and women. Curr Opin Psychiatry 23: 205–209. 20308902 10.1097/YCO.0b013e32833864eb PMC3784346

[36] Fisher JC, Bang H, Kapiga SH (2007) The association between HIV infection and alcohol use: a systematic review and meta-analysis of African studies. Sex Transm Dis 34: 856–863. 18049422 10.1097/OLQ.0b013e318067b4fd

[37] Kalichman SC, Simbayi LC, Vermaak R, Jooste S, Cain D (2008) HIV/AIDS risks among men and women who drink at informal alcohol serving establishments (Shebeens) in Cape Town, South Africa. Prev Sci 9: 55–62. 18264762 10.1007/s11121-008-0085-x

[38] Watt MH, Aunon FM, Skinner D, Sikkema KJ, Kalichman SC, et al (2012) “Because he has bought for her, he wants to sleep with her”: alcohol as a currency for sexual exchange in South African drinking venues. Soc Sci Med 74: 1005–1012. 22326304 10.1016/j.socscimed.2011.12.022 PMC3298605

[39] Papas RK, Sidle JE, Martino S, Baliddawa JB, Songole R, et al (2010) Systematic cultural adaptation of cognitive-behavioral therapy to reduce alcohol use among HIV-infected outpatients in western Kenya. AIDS and behavior 14: 669–678. 19967441 10.1007/s10461-009-9647-6 PMC2949418

[40] U.S. National Institutes of Health (2012) A Stage 2 Cognitive-behavioral Trial: Reduce Alcohol First in Kenya Intervention (RAFIKI). ClinicalTrialsgov.

[41] Morris M, Kretzschmar M (1995) Concurrent partnerships and transmission dynamics in networks. Soc Networks 17: 299–318.

[42] World Health Assembly (2007) Evidence-based strategies and interventions to reduce alcohol-related harm. Global assessment of public-health problems caused by harmful use of alcohol. Geneva: World Health Organization.

[43] World Health Assembly (2010) Strategies to reduce the harmful use of alcohol: draft global strategy. Geneva: World Health Organization.

[44] Motsoeneng T (2012) Insight: African alcohol binge raises pressure for crackdown. Reuters. Worcester, South Africa: Thomson Reuters.

[45] World Health Organization (2011) United Republic of Tanzania. Global Status Report on Alcohol and Health. Geneva.

[46] Department of Mental Health and Substance Abuse (2004) Global Status Report on Alcohol. Geneva: World Health Organization.

[47] Greene ME, Levack A (2010) Synchronizing Gender Strategies: A Cooperative Model for Improving Reproductive Health and Transforming Gender Relations. Population Reference Bureau.

[48] Pronyk PM, Kim JC, Abramsky T, Phetla G, Hargreaves JR, et al (2008) A combined microfinance and training intervention can reduce HIV risk behaviour in young female participants. Aids 22: 1659–1665. 18670227 10.1097/QAD.0b013e328307a040

[49] Feldman BS, Zaslavsky AM, Ezzati M, Peterson KE, Mitchell M (2009) Contraceptive use, birth spacing, and autonomy: an analysis of the Oportunidades program in rural Mexico. Stud Fam Planning 40: 51–62. 10.1111/j.1728-4465.2009.00186.x 19397185

[50] Hernandez-Prado B, Salomon JEU, Villalobos MDR, Figueroa JL (2005) Impact of Oportunidades on the Reproductive Health of Its Beneficiary Population. Cuernavaca: Instituto Nacional de Salud Publica.

[51] Steklov G, Winters P, Todd J, Regalia F (2006) Demographic Externalities from Poverty Programs in Developing Countries: Experimental Evidence from Latin America. Department of Economics Working Paper Series. Washington, D.C.: American University.

[52] Huerta MC, Hernandez D (2000) Algunos aspectos de salud reproductiva de la población beneficiaria de Progresa. In: PROGRESA: Mas oportunidades para las familias pobres. Evaluacion de resultados del Programa de Educacion, Salud y Alimentacion. Mexico City: Secretarıa de Desarrollo Social. 43–80.

[53] Sosa-Rubi SG, Walker D, Servan E, Bautista-Arredondo S (2011) Learning effect of a conditional cash transfer programme on poor rural women’s selection of delivery care in Mexico. Health policy and planning 26: 496–507. 21278371 10.1093/heapol/czq085 PMC9109227

[54] Gertler P (2004) Do Conditional Cash Transfers improve child health? Evidence from PROGRESA’s control randomized experiment. American Economic Review 94: 336–341. 29068185 10.1257/0002828041302109

[55] Rivera JA, Sotres-Alvarez D, Habicht JP, Shamah T, Villalpando S (2004) Impact of the Mexican program for education, health, and nutrition (Progresa) on rates of growth and anemia in infants and young children: a randomized effectiveness study. JAMA 291: 2563–2570. 15173147 10.1001/jama.291.21.2563

[56] Food and Agriculture Organization of the United Nations (2003) HIV/AIDS and Agriculture: Impacts and Responses: Case studies from Namibia, Uganda and Zambia. Rome.

[57] Shapouri S, Rosen S (2001) Toll on Agriculture from HIV/AIDS in Sub-Saharan Africa. Washington, D.C.: United States Department of Agriculture.

[58] Economic Commission for Africa (2006) Mitigating the impact of HIV/AIDS on smallholder agriculture, food security and rural livelihoods in Southern Africa: Challenges and action plan. Addis Ababa, Ethiopia.

[59] Oyefara JL (2007) Food insecurity, HIV/AIDS pandemic and sexual behaviour of female commercial sex workers in Lagos metropolis, Nigeria. Sahara J 4: 626–635. 18071614 10.1080/17290376.2007.9724884

[60] Dunkle KL, Jewkes RK, Brown HC, Gray GE, McIntryre JA, et al (2004) Transactional sex among women in Soweto, South Africa: prevalence, risk factors and association with HIV infection. Soc Sci Med 59: 1581–1592. 15279917 10.1016/j.socscimed.2004.02.003

[61] Agarwal B (1997) Bargaining and gender relations: Within and beyond the household. Feminist Economics 3: 1–51.

[62] United States Agency for International Development (2009) Land Tenure, Property Rights, and HIV/AIDS.

[63] The World Bank (2007) Agriculture for Development. Washington, D.C.

[64] United Nations Population Fund, The Center for International Earth Science Information Network (2007) Women’s Economic Empowerment: Meeting the Needs of Impoverished Women. New York, New York.

